# Teacher Well-Being: Teachers’ Goals and Emotions for Students Showing Undesirable Behaviors Count More Than That for Students Showing Desirable Behaviors

**DOI:** 10.3389/fpsyg.2022.842231

**Published:** 2022-04-28

**Authors:** Markus Forster, Christof Kuhbandner, Sven Hilbert

**Affiliations:** Department of Human Sciences, University of Regensburg, Regensburg, Germany

**Keywords:** teacher goals, teacher emotions, teacher well-being, negativity bias, occupational well-being

## Abstract

Previous findings indicate that the goals of teachers and their experienced emotions when interacting with students play an important role for their well-being. However, studies on the psychological impact of events have shown that the impact of bad events is stronger than the impact of good events. Thus, it may be that teachers’ goals and emotions for students showing undesirable behaviors (e.g., students who disrupt the class, do not finish their work, and have a negative attitude to learning) contribute more to their well-being than teachers’ goals and emotions for students showing desirable behaviors (e.g., students who pay attention in class, do their work on time, and have a positive attitude to learning), a distinction that has not been made in previous research. To examine this question, we measured teachers’ goals and emotions for students showing desirable and undesirable behaviors, and their affective, evaluative, occupational, and psychological well-being (*N* = 250). The results showed that teachers’ well-being was relatively strongly related to their goals and emotions for students showing undesirable behaviors: The higher the goals and the more positive the emotions, the higher the reported well-being. By contrast, the goals and emotions for students showing desirable behaviors were unrelated to teachers’ well-being. These results demonstrate that the principle of “bad is stronger than good” holds also for the influence of teachers’ goals and emotions on their well-being.

## Introduction

The essential role of teacher well-being in education has been increasingly recognized across the last years (McCallum et al., 2017). Based on the view that well-being of teachers is not only the absence of negative emotions and stress but also the presence of positive emotions and personal and professional flourishing (Huppert, 2009; Kern et al., 2014), it has been shown that teacher well-being has a positive impact on students’ learning and development. For instance, teacher well-being has been shown to be associated with higher academic gains of students (Caprara et al., 2003; Briner and Dewberry, 2007; Duckworth et al., 2009), to be an important precondition for the improvement of the well-being of students (Coleman, 2009; McCallum and Price, 2010; Sisask et al., 2014), the quality of teaching (for a review, see Hascher and Waber, 2021), and to enhance the professional motivation of teachers (McCallum, 2020). Experiencing well-being is also crucial for teachers themselves. Beyond the fact that well-being is a desirable good in itself, well-being is a precondition for teachers to experience teaching as a rewarding profession involving meaningful and important work, which prevents for the danger of loss to the profession (Pillay et al., 2005).

Given the important role of teacher well-being in education, recent studies have tried to identify factors that influence the well-being of teachers both at the level of general school conditions, such as job crafting (Dreer, 2022) and trust in colleagues (Huang et al., 2019), and the level of teacher-student interactions, such as student behavior (Aloe et al., 2014; Aldrup et al., 2018), experienced emotions when working with students (Spilt et al., 2011), or teacher goals (Rüprich and Urhahne, 2015), for reviews see (Acton and Glasgow, 2015; McCallum et al., 2017).

However, there is a basic principle of psychological functioning that may play an important role regarding the influence of many factors but has been neglected in the research on teacher well-being. Across a broad range of psychological phenomena, it has been found that the psychological effects of bad events (e.g., failures, being rejected by others, and receiving criticism) outweigh those of good events (e.g., success, being valued by others, and receiving praise), a principle that has been summarized in the often-cited quotation “bad is stronger than good” (Baumeister et al., 2001, p 323; for reviews, see Baumeister et al., 2001, Vaish et al., 2008).

This psychological principle may also play an important role for the experienced well-being of teachers. In their daily work, teachers are confronted with both good and bad events, experiencing teaching sometimes as influential, meaningful, and emotionally rewarding but sometimes also as uninfluential, meaningless, and emotionally stressful. However, despite of having both good and bad teaching experiences, the experienced well-being may be negatively biased due to the principle that bad experiences are given more weight than good experiences. For instance, although a teacher may actually successfully reach her/his educational goals for a large number of students and experience positive emotions when doing so, her/his well-being may nevertheless be low as soon as there are a few students for whom educational goals are difficult to reach and experienced emotions are negative.

The principle that bad is stronger than good has already been proven in a variety of fields, such as interpersonal interaction (Gottman, 1994), decision making (Kahneman and Tversky, 1979), learning (Miller, 1944), and teacher-student feedback (Coleman et al., 1987). However, while it has been shown that teachers´ goals and emotions when interacting with students do generally play a role for teacher well-being (e.g., Rüprich and Urhahne, 2015; Dreer, 2021), the question of whether teachers´ goals and emotions for students showing undesirable behaviors (“bad”) count more for teacher well-being than teachers´ goals and emotions for students showing desirable behaviors (“good”) has largely been neglected in the field of teacher research.

The aim of the present study was to examine whether the principle of bad is stronger than good plays a role in the experienced well-being of teachers. To examine this question, we measured the goals and experienced emotions of teachers for students showing desirable behaviors (i.e., students who pay attention in class, do their work on time, are well organized, and have a positive attitude to learning) and students showing undesirable behaviors (i.e., students who disrupt the class, do not finish their work, are unorganized, and have a negative attitude to learning), and determined the contributions of teachers’ goals and emotions for students showing desirable vs. undesirable behaviors to the teachers’ well-being.

Following previous operationalizations of well-being as a multifaceted construct encompassing elements of different psychological processes (Keyes, 2002; Seligman, 2012), teacher well-being was measured in terms of experienced positive and negative emotions (i.e., affective well-being), satisfaction with life (i.e., evaluative well-being; Diener et al., 1999), personal flourishing (i.e., psychological well-being; Ryff and Keys, 1995), and occupational functioning (i.e., occupational well-being; Van Horn et al., 2004). Whereas the experienced emotions of teachers are one of the main factors contributing to their affective well-being, the goals of teachers are one of the main factors determining their teaching-related evaluative, psychological, and occupational well-being. In fact, measuring only affective well-being without taking into account goal-related well-being may provide an incomplete picture of teacher well-being because a teacher may experience a high degree of affective well-being despite having dysfunctional goals and thus suboptimal psychological and occupational well-being.

## Materials and Methods

### Participants

In total, 250 secondary German teachers (165 women and 85 men) voluntarily participated in the study. The sample was recruited *via* advertisements at schools, social media, and personal contacts. To increase the motivation to participate, all participants received automated personal feedback at the end, and they could take part in a raffle to win vouchers worth 30 euros. The mean age of teachers was 44.39 years (ranging from 23 to 66 years, *SD* = 10.63), and on average, they have been working as a teacher for 15.85 years (ranging from 1 to 43 years, *SD* = 9.67). Most of the teachers worked full-time (64.0%) or part-time (34.8%). A 24% of them taught in lower track schools, 16.4% in intermediate track schools, 46.4% in comprehensive schools, 9.6% in technical college or higher vocational school, and 3.6% in other secondary schools. Reports on the teaching subjects revealed that German was most frequent (*n* = 83; 33.2%), followed by mathematics (*n* = 70; 28.0%), English (*n* = 60; 24.0%), history (*n* = 40; 16.0%), sport (*n* = 28; 11.2%), biology (*n* = 26; 10.4%), and a distribution across other subjects (e.g., chemistry, economy and law, physics, geography, music, French, and art; most of the teachers taught more than one subject). Although the sample was not designed to represent all teachers in Germany, a comparison with official statistical data (Statistical Offices of the Federal and State Governments, 2020; Federal Office of Statistics, 2022) showed that the sample characteristics closely match the distribution of demographical data for teachers in Germany regarding gender, age, working time, and subjects taught.

The study was conducted in accordance with the Helsinki Declaration and the University Research Ethics Standards of the University of Regensburg. All participants provided written informed consent. In Germany, these types of psychological studies do not require ethical approval of an Ethics Committee (see https://www.dfg.de/foerderung/faq/geistes_sozialwissenschaften/).

### Material and Procedure

Self-report data were collected using the online platform SoSci Survey (Leiner, 2018). The study consisted of two phases. In the first phase, participants’ affective, evaluative, psychological, and occupational well-being was measured, using well-established questionnaires (see below). Directly afterward, the second phase followed in which the participants’ goals and experienced emotions for students showing desirable behaviors and students showing undesirable behaviors were measured. Participants were instructed to put themselves mentally in the situation of a new school year where they will meet a new class of 16 students. They were told that the class will contain two types of students. To avoid an oversimplification, it was emphasized that the distinction of two types of students is an oversimplification, and that in reality far more complex manifestations and mixed forms of these simplified types are found, and that we do not claim that there could be any kind of an “ideal student.” To avoid conceptual priming effects, the types of students were neutrally labeled as “type 1” and “type 2.” The exact instruction was (original in German):

“Please mentally put yourself in the situation that a new school year is beginning. On the next page you will get to know your new students briefly, who exhibit personalities which you may have already experienced in a similar way in your real teaching life. In order to simplify the presentation, in the following, a distinction is made between two types of students. We are aware that in school practice there are far more complex manifestations and mixed forms of these simplified types, and we do not claim that there could be any kind of “ideal student.” Type I students are those who follow the lessons and participate. You can describe them with the adjectives diligent, ambitious, active thinking, or interested. Type II students are those who are less likely to follow or participate in class. They can be described with the adjectives lively, dominant, behaviorally problematic, aggressive, hyperactive, or disinterested.”

To introduce the class, participants were first shown the eight pictures of the students showing desirable behaviors at the same time for 30 s and second the eight pictures of the students showing undesirable behaviors at the same time for 30 s. The development of the photographs of the two types of students was based on a study by Hörstermann et al. (2010) where a cluster analysis of teacher students’ cognitive representation of student types is reported which revealed 10 clusters of student types. One of the clusters describes a type showing desirable behaviors (cluster description of behavioral characteristics: cooperating, working fast, attentive, concentrated, neat appearance, high performing, diligent, helpful, interested, and motivated) and the other nine clusters describe types showing undesirable behaviors (cluster descriptions of behavioral characteristics: talking a lot, dominant, cheeky, disruptive, attention seeking, aggressive, hyperactive, dreamy, lazy, uninterested, unmotivated, and insecure). Based on these descriptions, eight photographs of students showing desirable behaviors in a classroom situation and eight photographs of students showing undesirable behaviors in a classroom situation were developed. To control for possible effects of gender, photographs of four male and four female students were used for each of the types. The students wore neutral blue or gray clothing and were photographed against the same background. A detailed description of the individual photographs can be found at https://osf.io/jbsqf/ (document: description photographs of students).

After the introduction of all students, participants’ educational goals were measured separately for students showing desirable and undesirable behaviors using an established questionnaire (see below). Before working on the respective questionnaires, participants were shown the respective students a second time until participants pressed a button in order to start the questionnaire. Subsequently, experienced emotions for the students were measured. Participants were shown the photographs of the 16 students for 5 s each in random order. After each presentation, their experienced emotions were measured using a combined version of the affect grid (Russell et al., 1989) and the self-assessment manikin (Bradley, 1994; for details, see below).

### Measures

#### Affective Well-Being

Affective well-being was measured using the German version (Breyer and Bluemke, 2016) of the Positive and Negative Affect Schedule (PANAS; Watson et al., 1988), a self-report measure consisting of 10 positive (e.g., “enthusiastic”), and 10 negative adjectives (e.g., “distressed”). Participants responded on a five-point Likert scale ranging from 1 (not at all) to 5 (extremely) to describe how often they usually are in the respective emotional states. In the present sample, reliability on the 10 positive and negative items was high (Cronbach’s alphas = 0.84/0.86).

#### Evaluative Well-Being

Evaluative well-being was measured using the German version (Glaesmer et al., 2011) of the Satisfaction With Life Scale (SWLS; Diener et al., 1985), a self-report measure consisting of five statements reflecting a positive evaluation of one’s life quality (e.g., “I am satisfied with my life.”). Participants responded on a seven-point Likert scale ranging from 1 (strongly disagree) to 7 (strongly agree). In the present sample, reliability was high (Cronbach’s alphas = 0.85).

#### Psychological Well-Being

Psychological well-being was measured using the 18-item version of Ryff’s Psychological Well-Being Scale (PWB; Ryff et al., 2010), a self-report measure consisting of 18 statements reflecting the six areas of psychological well-being (the statements were adapted so that they referred to the context of teaching): autonomy (e.g., “I judge myself as a teacher by what I think is important, not by the values of what others think is important.”), environmental mastery (e.g., “I am good at handling the professional responsibilities of everyday life as a teacher.”), personal growth (e.g., “I think it is important to have new teaching experiences that challenge how I think about myself and the world.”), positive relation with others (e.g., “At school, I am perceived as a giving person, willing to share my time with others.”), purpose in life (e.g., “Some teachers wander aimlessly through life, but I am not one of them.”), and self-acceptance (e.g., “I like most parts of my personality.”). The original 54-item version of Ryff’s PWB questionnaire (Ryff, 1989) has been translated into German by Risch (2008), and the 18 items corresponding to the 18-item version of the questionnaire were used. Participants responded on a scale ranging from 1 (strongly disagree) to 7 (strongly agree). The total score was computed as the mean across all items. In the present sample, reliability was high (Cronbach’s alphas = 0.76).

#### Occupational Well-Being

Occupational well-being was measured using the job satisfaction scale of the Subjective Aspects of the Teaching Profession questionnaire (Dann et al., 2014), a self-report measure consisting of 12 statements (e.g., “I really enjoy my work as a teacher”). Participants responded on a four-point Likert scale ranging from 1 (does not apply to me in any way) to 4 (applies to me completely). In the present sample, reliability was high (Cronbach’s alphas = 0.85).

#### Teacher Goals

The participants’ goals for students showing desirable and undesirable behaviors were measured using the four student-related scales of the Questionnaire for the Assessment of Teacher Goals (FELZ; Rüprich and Urhahne, 2015), consisting of the scales consideration of individual differences, student engagement, relationship with students, and learning impact, a self-report measure consisting of four statements per scale. The statements were adapted so that they referred to either the group of the previously shown students showing desirable and undesirable behaviors [e.g., “In my job as a teacher, I strive to promote this type of students individually” (scale consideration of individual differences); “In my job as a teacher, I strive to hold interesting lessons for this type of students” (scale student engagement); “In my job as a teacher, I strive to build a trusting relationship with this type of students” (scale relationship with students); and “In my job as a teacher, I strive to be a teacher from whom this type of students learn a lot” (scale learning impact)]. Participants responded on a five-point Likert scale ranging from 1 (I do not agree) to 5 (I agree completely). The total score was computed as the mean across all items. In the present sample, reliability was estimated to be high (Cronbach’s alpha = 0.94).

#### Experienced Emotions

**Figure 1:**
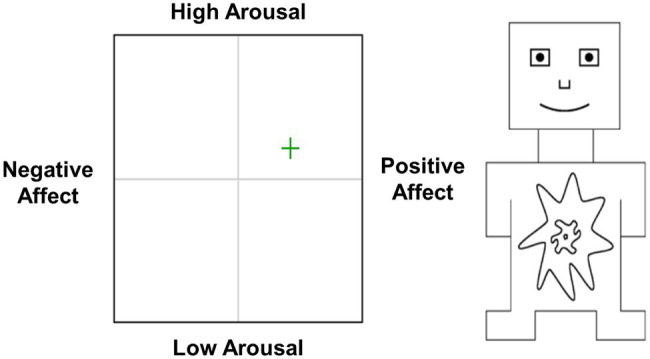
Illustration of the measurement of experienced emotions. An affect grid was shown (left side) which assesses experienced emotions on the dimensions of valence and arousal. Participants could move a cross across the grid, which resulted in respective changes in the manikin shown on the right side of the grid.

Experienced emotions were measured using a combined version of the affect grid (Russell et al., 1989) and the self-assessment manikin (Bradley, 1994). As depicted in Figure 1, an affect grid was shown on the screen which assesses experienced emotions on the dimensions of valence and arousal. Participants could move a cross across the grid, which resulted in respective changes in the manikin shown on the right side of the grid. That is, moving the cross along the valence axis changed the figure from frowning (negative) to smiling (positive), and moving the cross along the arousal axis changed the figure from eyes wide open and an explosive-like burst over the abdomen (high arousal) to eyes closed and a small pin prick over the abdomen (low arousal). The position of the cross on the grid on the valence dimension was converted in a valence score valence (−100 = extremely negative and +100 = extremely positive) and an arousal score (−100 = extremely low arousal and +100 = extremely high arousal).

## Results

Table 1 shows the means and SDs for the measured variables as well as the correlations between all variables. Teacher goals for students showing desirable and undesirable behaviors did not differ, *t*(249) = 0.4, *p* = 0.655, *d* = 0.03. Experienced emotions were more negative for students undesirable behaviors than for students showing desirable behaviors, *t*(249) = 31.5, *p* < 0.001, *d* = 1.99, and more arousing for students showing desirable behaviors than for students showing undesirable behaviors *t*(249) = 2.6, *p* = 0.010, *d* = 0.16.

**Table 1:** Correlations and descriptive statistics.

	1	2	3	4	5	6	7	8	9	10	11
1. Affective well-being (positive affect)		**−0.18** ^**^	**0.43** ^**^	**0.49** ^**^	**0.51** ^**^	**0.23** ^**^	**0.35** ^**^	0.06	**0.13** ^*^	0.00	0.02
2. Affective well-being (negative affect)			**−0.41** ^**^	**−0.45** ^**^	**−0.36** ^**^	**−0.13** ^*^	**−0.23** ^**^	−0.03	−0.12	0.05	0.02
3. Evaluative well-being				**0.44** ^**^	**0.45** ^**^	**0.16** ^*^	**0.17** ^**^	0.11	0.11	−0.02	0.05
4. Psychological well-being					**0.70** ^**^	**0.34** ^**^	**0.46** ^**^	0.02	**0.20** ^**^	−0.02	**0.13** ^*^
5. Occupational well-being						**0.15** ^*^	**0.29** ^**^	0.10	**0.14** ^*^	0.00	0.06
6. Goals (desirable behaviors)							**0.66** ^**^	0.06	0.06	**0.23** ^**^	0.11
7. Goals (undesirable behaviors)								0.02	**0.21** ^**^	0.03	**0.14** ^*^
8. Experienced emotional valence (desirable behaviors)									−0.09	0.12	**0.15** ^*^
9. Experienced emotional valence (undesirable behaviors)										0.04	−0.03
10. Experienced emotional arousal (desirable behaviors)											−0.02
11. Experienced emotional arousal (undesirable behaviors)											
*M*	3.51	1.60	5.52	5.46	3.06	4.05	4.04	54.46	26.53	−19.47	18.68
*SD*	0.56	0.54	0.94	0.60	0.48	0.51	0.64	20.14	33.42	29.39	33.70

*p* values are not corrected for multiple testing. Significant correlations are printed in bold.

* Indicates *p* < 0.05;

** Indicates *p* < 0.01.

**Figure 2:**
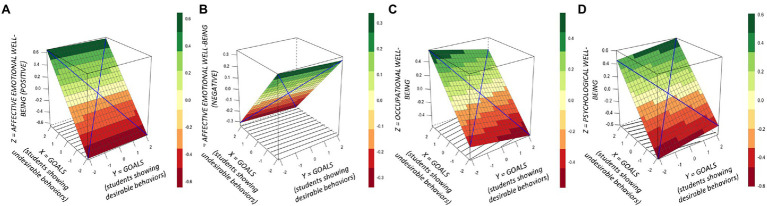
Response surface analysis plots: Links between teacher goals for students showing desirable and undesirable behaviors on **(A)** affective well-being (positive affect), **(B)** affective well-being (negative affect), **(C)** occupational, and **(D)** psychological well-being. The vertical *Z*-axis in the 3D figures refers to the level of well-being on a scale of −0,4 to 0,4 **(A)**; −0,3 to 0,3 **(B)**; −0,6 to 0,6 **(C)**; and −0,6 to 0,6 **(D)**. The higher the value, the higher the level of well-being. The color chart next to each figure denotes the numerical implication of the different hues. The *X*- and *Y*-axes reflect the values in experienced emotions for students showing desirable and undesirable behaviors, respectively. All values are standardized through *z*-transformations.

**Figure 3:**
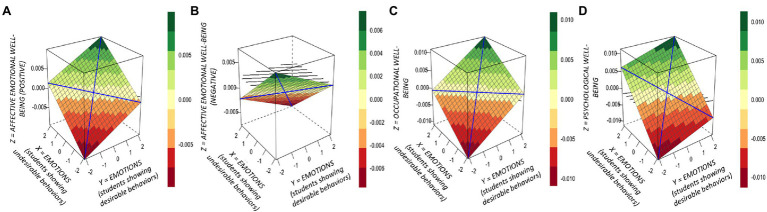
Response surface analysis plots: Links between teacher emotions (experienced emotional valence) for students showing desirable and undesirable behaviors on **(A)** affective well-being (positive affect), **(B)** affective well-being (negative affect), **(C)** occupational, and **(D)** psychological well-being. The vertical *Z*-axis in the 3D figures refers to the level of well-being on a scale of −0,005 to 0,005 **(A)**; −0,005 to 0,005 **(B)**; −0,010 to 0,010 **(C)**; and −0,010 to 0,010 **(D)**. The higher the value, the higher the level of well-being. The color chart next to each figure denotes the numerical implication of the different hues. The *X*- and *Y*-axes reflect the values in experienced emotions for students showing desirable and undesirable behaviors, respectively. All values are standardized through *z*-transformations.

To examine the effects of teacher goals and emotions on teacher well-being, multiple regression analyses were conducted with affective (positive and negative affect), evaluative, psychological, and occupational well-being as the dependent variables and (1) teacher goals for students showing desirable and undesirable behaviors as independent variables, and (2) teacher emotions (valence and arousal) for students showing desirable and undesirable behaviors as independent variables. Results are shown in Table 2 (effect of teacher goals) and Table 3 (effect of teacher emotions). The results depicted in Tables 2, 3 show, as illustrated in Figure 2 (effect of teacher goals) and Figure 3 (effect of teacher emotions), that affective, psychological, and occupational well-being depended on the height of teachers’ goals and experienced emotions for students showing undesirable behaviors. Descriptively, the effect was stronger for teacher goals than for teacher emotions. By contrast, neither teachers’ goals nor experienced emotions for students showing desirable behaviors did influence any of the well-being measurements.

**Table 2:** Results of regression analyses predicting level of affective, evaluative, psychological, and occupational well-being from teacher goals for students showing desirable and undesirable behaviors.

Measure	*B*	95% CI		*SE B*	*β*	*t*	*p*
		*LL*	*UL*				
Affective well-being (positive affect; *R*^2^ = *R*^2^*_adj_* = 0.12)							
Goals (desirable behaviors)	−0.01	−0.18	0.16	0.09	−0.01	−0.13	0.89
Goals (undesirable behaviors)	0.31	0.17	0.45	0.07	0.36	4.47	<0.001
Affective well-being (negative affect; *R*^2^ = *R*^2^*_adj_* = 0.05)							
Goals (desirable behaviors)	0.04	−0.13	0.21	0.09	0.04	0.46	0.65
Goals (undesirable behaviors)	−0.22	−0.36	−0.08	0.07	−0.25	−3.07	0.002
Evaluative well-being (*R*^2^ = *R*^2^*_adj_* = 0.03)							
Goals (desirable behaviors)	0.16	−0.14	0.47	0.15	0.09	1.06	0.29
Goals (undesirable behaviors)	0.16	−0.08	0.41	0.12	0.11	1.33	0.19
Psychological well-being (*R*^2^ = 0.21; *R*^2^*_adj_* = 0.20)							
Goals (desirable behaviors)	0.07	−0.11	0.25	0.09	0.06	0.77	0.44
Goals (undesirable behaviors)	0.40	0.25	0.54	0.07	0.42	5.52	<0.001
Occupational well-being (*R*^2^ = 0.09; *R*^2^*_adj_* = 0.08)							
Goals (desirable behaviors)	−0.07	−0.22	0.08	0.08	−0.07	−0.90	0.37
Goals (undesirable behaviors)	0.26	0.14	0.38	0.06	0.34	4.19	<0.001

CI, confidence interval; LL, lower limit; UL, upper limit; *β*, standardized regression coefficient; *t*, *t*-value; *p*, probability of committing a Type-I-Error; and *p* values are not corrected for multiple testing.

**Table 3:** Results of regression analyses predicting level of affective, evaluative, psychological, and occupational well-being from experienced emotional valence and arousal for students showing desirable and undesirable behaviors.

Measure	*B*	95% CI		*SE B*	*β*	*t*	*p*
		*LL*	*UL*				
Affective well-being (positive affect; *R*^2^ = 0.02; *R*^2^*_adj_* = 0.01)							
Experienced emotional valence (desirable behaviors)	0.002	−0.002	0.01	0.002	0.07	1.07	0.29
Experienced emotional valence (undesirable behaviors)	0.003	0.00	0.01	0.001	0.14	2.22	0.03
Experienced emotional arousal (desirable behaviors)	0.00	−0.002	0.002	0.001	−0.01	−0.16	0.87
Experienced emotional arousal (undesirable behaviors)	0.00	−0.002	0.002	0.001	0.01	0.22	0.82
Affective well-being (negative affect; *R*^2^ = 0.02; *R*^2^*_adj_* = 0.01)							
Experienced emotional valence (desirable behaviors)	−0.001	−0.01	0.002	0.002	−0.05	−0.82	0.41
Experienced emotional valence (undesirable behaviors)	−0.002	−0.01	0.00	0.001	−0.13	−1.99	0.05
Experienced emotional arousal (desirable behaviors)	0.001	−0.001	0.003	0.001	0.07	1.02	0.31
Experienced emotional arousal (undesirable behaviors)	0.00	−0.002	0.002	0.001	0.02	0.36	0.72
Evaluative well-being (*R*^2^ = 0.03; *R*^2^*_adj_* = 0.01)							
Experienced emotional valence (desirable behaviors)	0.01	0.00	0.01	0.003	0.12	1.88	0.06
Experienced emotional valence (undesirable behaviors)	0.004	0.00	0.01	0.002	0.12	1.94	0.05
Experienced emotional arousal (desirable behaviors)	−0.001	−0.01	0.002	0.002	−0.04	−0.66	0.51
Experienced emotional arousal (undesirable behaviors)	0.001	−0.003	0.004	0.002	0.03	0.54	0.59
Psychological well-being (*R*^2^ = 0.06; *R*^2^*_adj_* = 0.04)							
Experienced emotional valence (desirable behaviors)	0.001	−0.003	0.004	0.002	0.02	0.31	0.76
Experienced emotional valence (undesirable behaviors)	0.004	0.002	0.01	0.001	0.20	3.26	0.001
Experienced emotional arousal (desirable behaviors)	−0.001	−0.003	0.002	0.001	−0.03	−0.51	0.61
Experienced emotional arousal (undesirable behaviors)	0.002	0.00	0.01	0.001	0.13	2.05	0.04
Occupational well-being (*R*^2^ = 0.03; *R*^2^*_adj_* = 0.02)							
Experienced emotional valence (desirable behaviors)	0.003	−0.001	0.01	0.002	0.10	1.62	0.11
Experienced emotional valence (undesirable behaviors)	0.002	0.00	0.01	0.001	0.15	2.36	0.02
Experienced emotional arousal (desirable behaviors)	0.00	−0.002	0.002	0.001	−0.02	−0.24	0.81
Experienced emotional arousal (undesirable behaviors)	0.001	−0.001	0.002	0.001	0.05	0.72	0.47

CI, confidence interval; LL, lower limit; UL, upper limit; *β*, standardized regression coefficient; *t*, *t*-value; *p*, probability of committing a Type-I-Error; and *p* values are not corrected for multiple testing.

## Discussion

Numerous findings have shown that the psychological impact of bad events is stronger than the psychological impact of good events. The present findings indicate that this holds also true for the well-being of teachers. Teachers’ affective, occupational, and psychological well-being were relatively strongly related to their goals and experienced emotions for students showing undesirable behaviors: the higher the goals and the more positive the experienced emotions, the higher the reported well-being. By contrast, the goals and experienced emotions for students showing desirable behaviors were unrelated to the teachers’ well-being.

The finding that teachers’ goals and emotions for students showing undesirable behaviors have a higher impact on teacher well-being than teachers’ goals and emotions for students showing desirable behaviors is in line with numerous findings demonstrating the principle of “bad is stronger than good” in a variety of psychological domains (Baumeister et al., 2001). The common explanation is that a higher psychological impact of bad events compared to good events is adaptive because the costs of failure of adequate responding to bad events can be much higher than the costs of failure of adequate responding to good events (Baumeister et al., 2001). For instance, while a failure to respond adequately to a snake can have fatal consequences, a failure to respond adequately to a pleasant object can possibly be corrected at the next attempt. Consistent with such a hypothesis, numerous studies have shown that bad objects are more efficiently and preferably processed (Ohman et al., 2001; Dijksterhuis and Aarts, 2003; Nasrallah et al., 2009; Kuhbandner et al., 2011).

In fact, a similar mechanism may explain the present finding that teachers’ goals and emotions for students showing undesirable behaviors have a higher impact on their well-being than teachers’ goals and emotions for students showing desirable behaviors. When judging their well-being, teachers may more strongly focus on their experiences with students showing undesirable behaviors so that their experienced well-being may be mainly determined by the experiences they make with these students. Experiencing more positive emotions when interacting with students showing undesirable behaviors and being able to pursue higher goals for these students will thus result in higher experienced well-being. Due to the focus on students showing undesirable behaviors, experiences with students showing desirable behaviors may contribute less to one’s experienced well-being, and even if a teacher experiences positive emotions and pursues high goals when interacting with students showing desirable behaviors, this may not matter when there are students showing undesirable behaviors who elicit negative emotions and for whom no high goals are pursued. That is, even if a teacher actually successfully achieves high goals for a large number of students and experiences positive emotions when doing so, the well-being of a teacher can still be low if there are some students for whom educational goals are difficult to achieve.

From an applied perspective, the present finding opens up new ways to increase the well-being of teachers. On the one hand, well-being may be increased by giving more weight to goals and emotions for students showing desirable behaviors when judging ones’ well-being. On the other hand, well-being may be increased by helping teachers to establish high goals and experience more positive emotions when interacting with students showing undesirable behaviors. To achieve the latter, it is important to make teachers aware that it is important to pursue high goals not only for students showing desirable behaviors but also for students showing undesirable behaviors, and to help them to develop skills to reach high goals for students showing undesirable behaviors. Furthermore, from a motivational perspective, it could be essential to supplement the most frequently mentioned motive for teaching “Because I like to work with children and adolescents” (Rothland, 2011) by the phrase “both with students showing desirable behaviors and with students showing undesirable behaviors.”

At first glance, the results of the present study seem to suggest that the impact of teacher goals on teacher well-being is stronger than the impact of teacher emotions. In fact, the effect of teacher emotions was small, and when correcting for multiple testing, the effect of teacher emotions for students showing undesirable behaviors on some of the well-being measures no longer reached conventional levels of significance. However, it is important to note that, although both goals and emotions were measured with reference to the same sets of specific pictures of students, goals and emotions were measured in different ways. Teacher goals were measured using a questionnaire which assesses one’s general goals for students showing desirable and undesirable behaviors (e.g., “In my job as a teacher, I strive to hold interesting lessons for this type of students”). By contrast, teacher emotions were assessed by measuring the participants’ experienced emotions for the specific set of students shown on the pictures. That is, whereas goals were measured in a more trait-like manner, emotions were measured in a more state-like manner. Since state measures are more prone to situational effect which may introduce stronger situation-specific variance, the observed stronger effects of goals compared to experienced emotions may reflect the fact that goals were measured more trait-like and emotions measured more state-like.

Another reason for the observed stronger effects of goals compared to experienced emotions may be that experienced emotions were measured using pictures of students showing desirable or undesirable behaviors. Emotional experiences may be stronger when elicited in real-life classroom situations and may thus play a larger role for teacher well-being as suggested by the measured emotional responses to pictures of classroom situations. Furthermore, another potential limitation of the present study is that the data were collected in an online environment which allows only limited control. Accordingly, further examining the relationship between the goals and emotions of teachers and their well-being in real-life situations may be an interesting avenue for future research.

There is a relatively large body of research indicating that goals (i.e., explicit motives) and emotions (i.e., implicit motives) are often largely unrelated (for a meta-analysis, see Köllner and Schultheiss, 2014). This is also supported by the present study where the height of goals and the valence of experienced emotions were uncorrelated for students showing desirable behaviors and only slightly correlated for students showing undesirable behaviors. Regarding the effects on well-being, there is evidence that a congruency between implicit and explicit motives is associated with elevated well-being (for a review, see Thrash et al., 2012). Accordingly, examining whether this may also be true for the relationship between teachers’ goals and emotions for students and their experienced well-being is an interesting research question. However, examining this question with the present data is problematic since, as described above, goals were measured in a more trait-like manner, whereas emotions were measured in a more state-like manner. Accordingly, it is difficult to draw valid conclusions about the interplay of goals and emotions based on the present data. Examining this issue is an interesting avenue for future research.

The present findings indicate that teachers’ goals and experienced emotions for students showing undesirable behaviors contribute more to their well-being than teachers’ goals and emotions for students showing desirable behaviors. An open question is, however, which factors may explain why teachers differ in the height of their goals for students showing undesirable behaviors. One possibility is that teachers with higher goals and more positive emotions for students showing undesirable behaviors have a higher ability to regulate the problematic behavior of these students. Another possibility is that teachers with higher goals and more positive emotions for students showing undesirable behaviors had less contact so far with students showing highly undesirable behaviors. In fact, such factors may contribute to the observed finding that teacher with higher goals and more positive emotions for students showing undesirable behaviors show higher well-being. Although examining this issue is an important avenue of future research, this does not concern the main finding of the present study that teachers’ goals and experienced emotions for students showing undesirable behaviors contribute more to their well-being than teachers’ goals and emotions for students showing desirable behaviors.

In conclusion, the present findings indicate that the psychological principle of “bad is stronger than good” holds also for the influence of teachers’ goals and emotions on their well-being. Whereas teachers’ goals and emotions for students showing undesirable behaviors had a relatively strong impact, their well-being was entirely independent of their goals and emotions for students showing desirable behaviors. From an applied perspective, it may thus be helpful to make teachers aware of the psychological principle of “bad is stronger than good,” and to help them to realize that the setting of high goals for students showing undesirable behaviors is an important ingredient for their teacher well-being.

## Data Availability Statement

The datasets presented in this study can be found in online repositories. The names of the repository/repositories and accession number(s) can be found at: https://osf.io/jbsqf/.

## Ethics Statement

Ethical review and approval was not required for the study on human participants in accordance with the local legislation and institutional requirements. The patients/participants provided their written informed consent to participate in this study.

## Author Contributions

MF and CK developed the research idea and drafted the manuscript. MF designed the study. MF, CK, and SH analyzed the data. All authors contributed to the article and approved the submitted version.

## Funding

The project which this article is part of is supported by the federal and state governments as part of the joint “Qualitätsoffensive Lehrerbildung” (Teacher Education Quality Offensive), which is funded from the Federal Ministry of Education and Research under the grant number 01JA1812. The authors are responsible for the content of this publication.

## Conflict of Interest

The authors declare that the research was conducted in the absence of any commercial or financial relationships that could be construed as a potential conflict of interest.

## Publisher’s Note

All claims expressed in this article are solely those of the authors and do not necessarily represent those of their affiliated organizations, or those of the publisher, the editors and the reviewers. Any product that may be evaluated in this article, or claim that may be made by its manufacturer, is not guaranteed or endorsed by the publisher.
